# Supplementary material for: Rate dependent neural responses of interaural-time-difference cues in fine-structure and envelope
Source: PeerJ. 2024 Apr 24;12:e17104. doi: 10.7717/peerj.17104 (PMC11055513; doi:10.7717/peerj.17104)
Supplement: Supplemental Information 1 [file peerj-12-17104-s001.pdf]

Supplementary Materials

Hongmei Hu [1,2,3*], Stephan D. Ewert[2,3], Birger Kollmeier[2,3], Deborah Vickers[1]

[1]SOUND Lab, Cambridge Hearing Group, Department of Clinical Neuroscience, Cambridge University, Cambridge, UK
[2]Department of Medical Physics and Acoustics, University of Oldenburg, Oldenburg, Germany
[3]Cluster of Excellence “Hearing4all”, University of Oldenburg, Oldenburg, Germany

Corresponding Author:
Hongmei Hu
Küpkersweg 74, 26129, Oldenburg, Germany
Email address: hh594@cam.ac.uk, hongmei.hu@uol.de

Materials & Methods

Test procedures

Psychoacoustic pretest-experiments

Twelve NH participants (S1 - S12: 6 males and 6 females aged 21- 42 years old, with a mean age of 27.2 years) took part in the psychoacoustic experiment. In the psychoacoustic experiments, the stimuli were generated digitally using a personal computer running MATLAB (The Mathworks, Natick, MA, USA), then converted to analog form using a Fireface UC sound card (RME Audio, Haimhausen, Germany) with 24-bit resolution and a sampling rate of 48 kHz. Then the stimuli were presented to the participants through Sennheiser HD580 headphones at 70 dB sound pressure level (SPL). The participants were seated in a double-walled soundproof booth and responded by clicking the virtual buttons displayed on a monitor.
Three lateralization experiments (5-7 minutes/experiment) were performed to determine the upper frequency limit of left/right discrimination abilities. The experiments used a two-up, one-down, two-alternative forced-choice procedure (2-AFC) to estimate the 71%-correct threshold on the psychometric function (Levitt, 1971). On each trial, two consecutive intervals were presented, separated by 500 ms. Each interval contained four consecutive 400-ms tones or filtered clicks, with 20-ms raised cosine rise/fall ramps, separated by 100 ms. One interval was randomly selected as the standard and had an ITD of the carrier ($\mathrm{ITD_{FS}}$) or ITD of the envelope ($\mathrm{ITD_{ENV}}$) of 0. The other interval, the target, had the same first and third tones as the standard, but the second and fourth tones had a non-zero ITD of the same magnitude as each other. During all three experiments, participants were asked to identify which of the two intervals contained a sequence that appeared to change within the head.
Experiment 1 was conducted to determine the upper limit of the carrier frequency ($f_{\mathrm{uplim\_}c}$) for fine structure ITD sensitivity by applying an IPD of π/2 to the carrier frequency. The experiment utilized SAM tones with a fixed modulation frequency (f_m) of 40 Hz and an adaptive carrier

frequency (f_c) ranging from 100 Hz to 4000 Hz. The carrier frequency was adjusted using adaptation factors of 1.4, 1.2, and 1.1, starting at 1000 Hz. Before the formal experiment, a brief training task was provided to familiarize the participants with the procedures. After eight reversals, the formal test was terminated and the threshold was calculated as the geometric mean of the last six reversal values. This procedure was adapted from the binaural TFS sensitivity test (TFS-AF) (Füllgrabe et al., 2017; Füllgrabe and Moore, 2017).

Experiment 2 was conducted to determine the upper limit of the modulation rate (f_{uplim_m}) for envelope ITD sensitivity, with a fixed ITD of 500 µs (dichotic) or 0 (diotic) applied to the envelope. The f_c was fixed at 4000 Hz, while the f_m was adaptive. Experiment 3 was performed to determine the upper limit of the pulse rate (f_{uplim_pps}) for interaural pulse time difference (IPTD) sensitivity, using an ITD of 500 µs applied to pulses and adjusting the pulse rate. Both experiments were similar to experiment 1, except that in experiments 2 and 3, the start f_m or pulse rate was 100 Hz or 100 pps, with a minimum and maximum of 10 Hz or pps and 1500 Hz or pps, respectively. The adaptation factors were 80, 40, and 20. To accommodate individual differences, an upper limit for the adaptive f_m was not set. This may have resulted in some participants perceiving lower sidebands of the modulation as audible for higher modulation rates (e.g., above ~350 Hz) (Kohlrausch et al., 2000), transforming the ITD change detection task into a disparity detection task not only based on envelope ITD cues. The decision not to set an upper limit was made to consider the possibility that participants may use other cues, which could also trigger ACC responses.

Stimuli

SAM tones

In both experiments 1 and 2, SAM tones were generated digitally according to equation (1) (Bernstein and Trahiotis, 2012; Hu et al., 2022).

$$s(t) = a\, sin(2\pi f_c\, t)(1 - cos2\,\pi f_m t) \qquad (1)$$

Figure 3A, column 1, shows an example of a stimulus used in the psychoacoustic experiment 1 (SAM tones ITD_{FS}, f_c = 1000 Hz and f_m= 40 Hz). In this example, the first interval is the target (row 2), and the second interval is the standard (row 3). Both the psychoacoustic and EEG experiments employed IPD of 0 or π/2. EEG experiment 1 tested four carrier frequencies, f_c = [400, 800, 1200, 1600] Hz. Figure 3B, column 1, shows an example of a stimulus used in EEG experiment 1 (SAM tones ITD_{FS}, f_c = 400 Hz and f_m= 40 Hz), where each presentation lasted 8 seconds (s). The sequence included 2 s of the diotic stimulus (IPD = 0 in time window T1), followed by 2 s of the dichotic stimulus (IPD = π/2 in time window T2; T1→T2 referred to as outward switching), then 2 s of the standard stimulus (IPD = 0 in time window T3; T2→T3 referred to as inward switching), and 2 s of silence (in time window T4).

The stimuli used in the second psychoacoustic experiment (not shown in Figure 3A) were also SAM tones, but the ITD was applied to the envelope instead of the carrier. In EEG experiment 2, four modulation frequencies were tested f_m = [40, 80, 160, 320] Hz. Figure 3B, column 2 shows an example of a stimulus used in this experiment (SAM tones ITD_{ENV}, f_c = 4000 Hz and f_m = 40 Hz). Like in EEG experiment 1, it consisted of 2 s of the diotic stimulus (ITD_{ENV} = 0 in the time window T1), followed by 2 s of the dichotic stimulus (ITD_{ENV} = 500 µs in the time

window T2; with an outward switching T1→T2), then again 2 s of the standard stimulus ($\mathrm{ITD_{ENV}}$ = 0 in the time window T3; with an inward switching T2→T3), and 2 s of silence (in the time window T4). As Ross (2018) showed no detectable ACCs in most of their participants for the 4000 Hz SAM, the ACCs in experiment 2 might be smaller than in experiment 1 or absent.
Note that, as in (Kohlrausch et al., 2000), no precautions were taken to mask possible distortion products in both psychoacoustic and the EEG experiment 2.

Filtered clicks

In experiment 3, filtered clicks generated as in (Hu et al., 2017; Hu et al., 2022) were used to simulate the signal delivered to CI users. The pulse train was band-limited to 3-5 kHz with a center frequency of f_c = 4 kHz. These band-limited pulse trains $p(t)$ were then sinusoidally amplitude-modulated using formula (2).

$$s(t) = p(t) * [1 - cos(2\pi * f_m * t)] \qquad (2)$$

The f_m in the psychoacoustic was 2.5 Hz (reciprocal of the duration of consecutive filtered clicks, i.e. 1/0.4s), while it was 10 Hz in EEG experiments. This type of SAM ensures that stimuli start at the trough of the modulation.
Figure 3A (column 2) and B (column 3) show an example of the stimuli used in the psychoacoustic (filtered clicks IPTD, pulse rate = 160 pps and f_m= 2.5 Hz, IPTD = 0 or IPTD = 500 µs) and EEG experiments (filtered clicks IPTD, pulse rate = 160 pps and f_m= 10 Hz, IPTD = 0 or IPTD = 500 µs), respectively. In the EEG experiment, four fixed pulse rates of [40, 80, 160, 320] pps were used. The duration of each presentation is 6 s, which includes 2 s of the diotic stimulus, followed by 2 s of the dichotic stimulus (with a transition from T1→T2, referred as outward switching), and 2 s of silence (in time window T4, with a T2→T3 inward switching).
In both the psychoacoustics and EEG experiment 3, a low-pass noise, uncorrelated between the ears, was added to the filtered clicks to conceal potential distortion products. The low-pass noise was created by generating broadband noise in the time domain, converting it to the frequency domain, and setting the power of all components above 1000 Hz to zero. The noise was then manipulated to have a flat spectrum up to 200 Hz with a decreasing spectral density of 3 dB/octave above 200 Hz. It was further filtered with a 5th-order, lowpass filter with a cut-off frequency of 1000 Hz (Hu et al., 2017), and gated with 50-ms raised cosine ramps. The test stimulus was centered within the noise presentation, which was presented at 40 dB SPL.
We chose 4000 Hz instead of a higher carrier frequency such as 8000 Hz for several reasons: Firstly, Previous studies have shown that the upper modulation rate is lower for stimuli centered at 8000 Hz compared to those centered at 4000 Hz (Bernstein and Trahiotis, 2013). Since only 2 out of 14 participants in Ross (2018) showed significant responses at 4000 Hz, we would expect similar or even smaller responses at 8000 Hz. Secondly, as the aging population is one target group for future studies, high-frequency hearing loss may make a higher frequency less optimal. Lastly, although it is not a critical factor, 8000 Hz is less pleasant to listen to than 4000 Hz.

Results

Psychoacoustic pretest results

Supplementary Figure 1 shows the violin plots of the f_{uplim_c}, f_{uplim_m}, and f_{uplim_pps} from three psychoacoustic experiments. The violin plots (Hintze and Nelson, 1998) were generated using freely available Matlab code (https://github.com/bastibe/Violinplot-Matlab). The original box plot shape is included as a grey box in the center of the violin. Supplementary Figure 1 depicts the individual data of the 12 participants as solid blue dots that have been randomly jittered from the center. The corresponding density curves have been constructed around each center line. If the participant couldn't do the task, the value was set to 0.123456. Supplementary Figure 1 indicates that the upper limits vary across participants. The mean and standard deviation of f_{uplim_c} is 1393 ± 284 Hz, which is in the range of previously reported values (Ross et al., 2007a; Ross et al., 2007b; Grose and Mamo, 2010; Hopkins and Moore, 2010, 2011; Brughera et al., 2013; Füllgrabe and Moore, 2017; Papesh et al., 2017; Füllgrabe and Moore, 2018). It should be noted that the top-performing participant in this study exhibited a higher f_{uplim_c} than those reported in (Klug and Dietz, 2022), possibly due to the utilization of different stimuli and test procedures. The purpose of the pretests is to select the rate conditions for in the EEG experiments. The exactly upper frequency limit in humans is not the focus of this study, and more detailed discussions are beyond the scope of this paper.

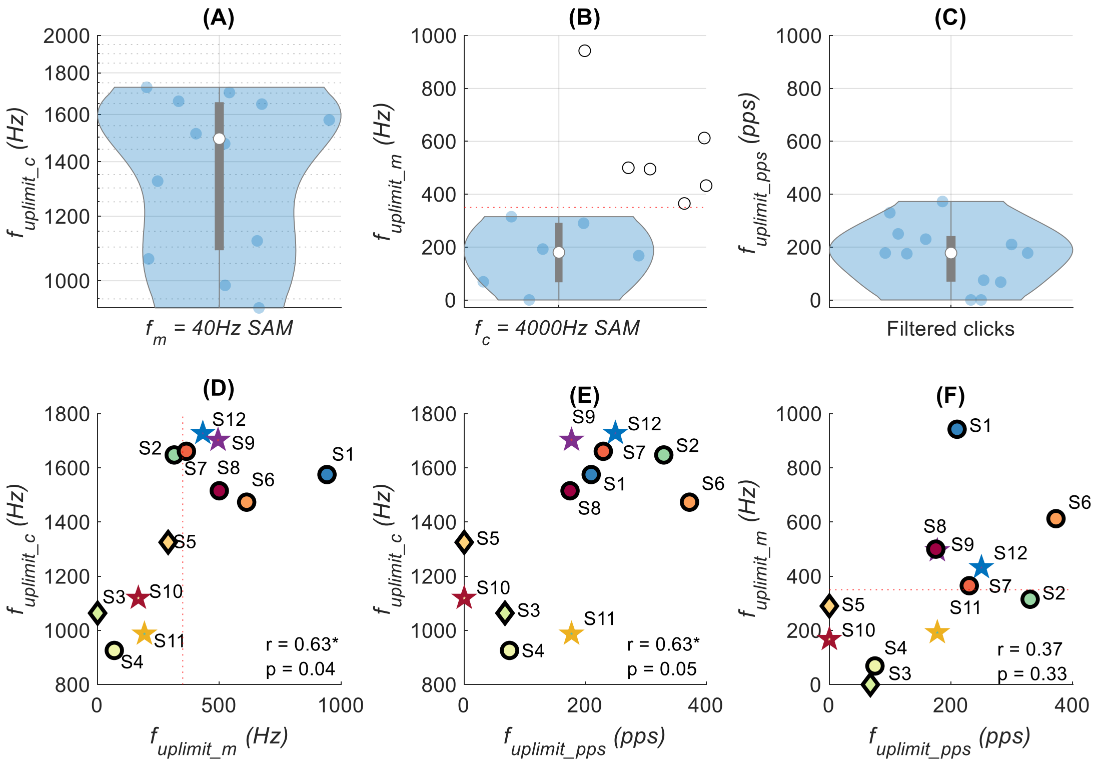

Supplementary Figure 1 the top panels of the figure show violin plots of the upper limit frequency f_c obtained from the psychoacoustic experiment for each participant, represented by solid dots in each violin plot. The bottom panels display the correlation between the three upper limit frequencies. Participants S9-S12 (represented by pentagram symbols) were unable to attend the EEG experiment, while S3 couldn't achieve f_{uplim_m} and S5 couldn't achieve f_{uplim_pps}, represented by diamond symbols. The dotted red lines in panels B, D, and F indicate the boundary of 350 Hz.

The top middle panel of Supplementary Figure 1 shows the f_{uplim_m}. Without setting a limit in the adaptive procedure, some participants reached f_{uplim_m} above 350 Hz. Participant S1 even reached 980 Hz. This was expected because the task may become easier again for some

participants if they are able to use spectrally resolve sidebands at modulation rates above a certain frequency (e.g., ~ 350 Hz, the red dotted horizontal line) (Kohlrausch et al., 2000). This phenomenon may be more prominent in the disparity detection test procedure used in this study, compared to the classical left/right discrimination tasks. To avoid misleading interpretation, the mean and standard deviation of f_{uplim_m} were calculated after excluding data from participants who couldn't complete the task (S3) and those with f_{uplim_m} above 350 Hz (6 data points as indicated by the empty circles in the upper middle panel of Supplementary Figure 1, which may be a result of the resolved sidebands). The resulting mean and standard deviation were 207 ± 99 Hz. Some caution is necessary when interpreting the correlation between the f_{uplim_m} and other experimental results. However, the same issue was not apparent for the EEG results shown in Section 3.2, because the maximum modulation frequency tested in the EEG experiment was limited to 320 Hz. The mean and standard deviation of f_{uplim_pps} for filtered clicks, after excluding S5 and S10, were 207 ± 97 pps. The Pearson correlation coefficients between the three upper limits are as follows: between f_{uplim_c} and f_{uplim_m} (exclude S3), r = 0.63, p = 0.04; between f_{uplim_c} and f_{uplim_pps} (excluded S5 and S10), r = 0.63, p = 0.05; and between f_{uplim_m} and f_{uplim_pps} (excluded S3, S5, and S10), r = 0.37, p = 0.33.

S3 and S5 were unable to perform the corresponding experiments. However, both detected changes when presented with 100 Hz SAM tones and 100 pps filtered clicks with 500 µs ITD. It was speculated that this was mainly due to the large initial adaptive stepsize and their difficulty in focusing during the first reversal. S8 reported that he occasionally experienced mild tinnitus. Despite this, he was included in the EEG experiment as his audiometry results were within normal hearing range and his lateralization performance was above average. Regrettably, participants S9-S12 were unable to attend the EEG experiments due to reasons relating to the COVID-19 pandemic.

Additional EEG results

Time domain (CAEPs)

- CAEPs of experiment 1

Regarding experiment 1 ($\mathrm{ITD_{FS}}$, pape Figure 4A and Figure 6A), the amplitude and latency of the offset responses (aqua) were relatively consistent across different carrier frequencies and the N1 latency of the offset responses was generally shorter compared to the onset and ACC responses.

The N1P2 amplitude was significantly affected by carrier frequency (f_c), response type, and their interaction according to GLMrm (p<0.005). The mean amplitude was 7.318/6.063/5.094/4.023 µV for 400/800/1200/1600 Hz, respectively. There were no significant differences between 400, 800, and 1200 Hz, but the N1P2 amplitude for the 1600 Hz was significantly smaller than the other carrier frequencies. For the offset CAEPs, there were no significant differences between carrier frequencies. For most of the onset CAEPs, the differences were not significant except that the N1P2 amplitude of 1200 Hz was slightly larger than that of 1600 Hz (p = 0.048). For ACC1 (outward) responses, the N1P2 amplitudes of 400

Hz and 800 Hz were significantly larger than the 1200 and 1600 Hz, but there were no significant differences between 400 and 800 Hz, and between 1200 Hz and 1600 Hz. For ACC2 (inward) responses, the N1P2 amplitude of 800 Hz was significant larger than that of 1200 and 1600 Hz, and the N1P2 amplitude of 400 Hz was significantly larger than that of 1600 Hz.

The mean N1P2 amplitudes were 8.577/4.816/4.172/4.932 µV for onset/ACC1/ACC2/offset responses, respectively. The onset CAEPs were significantly larger than the ACC1, ACC2, and offset CAEPs. However, there were no significant differences between the three latter types. Pairwise comparisons within each f_c showed that the onset CAEPs were significantly larger than the offset CAEPs only for 400 and 1200 Hz. There were no significant differences between ACC1 and ACC2 for all carrier frequencies. Significant correlations were observed between the onset N1P2 amplitudes of most carrier frequencies, except for 400 vs 1200 Hz, and 400 vs 1600 Hz.

The mean N1 latency was 114/132/137/95 ms for onset/ACC1/ACC2/offset, respectively. The GLMrm analysis showed a significant effect of response type ($p<0.001$), but no significant effect of f_c and their interaction. Pairwise comparisons showed significant differences between most response types ($p<0.01$), except between ACC1 and ACC2. The N1 latency of ACC responses was significantly larger than the onset response, while the offset response had the shortest latency and was significantly smaller than the other response types.

In summary, the results from experiment 1 were generally consistent with Ross et al. (2007b). For example, the mean P1, N1, and P2 amplitudes of ACC were smaller than those of the onset response: P1, 1.684/1.405/1.005/0.248 µV; N1, 3.324/-1.299/-1.019/-2.146 µV; P2, 3.946/2.128/1.862/2.379 µV for onset/acc1/acc2/offset. The ACC latencies were delayed compared with the corresponding onset and offset ones: P1, 42/46/57/27 ms; N1, 114/132/137/95 ms; P2, 211/227/240/213 ms for the onset/ACC1/ACC2/offset. The mean latencies of both P1 and N1 were in the same range but slightly smaller than Ross et al. (2007a). The latencies of $\mathrm{ITD_{FS}}$ change evoked ACC1 and ACC2 that were longer than the onset, and the differences were smaller than Ross et al. (2007a). Consistent with (Ross, 2018), there was a tendency for larger responses to outward IPD changes (ACC1) than inward changes (ACC2) for the lower carrier frequencies, however, it was not significant here ($p>0.5$).

- CAEPs of experiment 2

In experiment 2 ($\mathrm{ITD_{ENV}}$), as demonstrated in paper Figure 4B and Figure 6B, similar to experiment 1, there were clear onset and offset responses in all four test conditions. The onset N1P2 amplitude was comparatively larger than the offset responses, but the difference between the onset and offset CAEPs was smaller compared to those shown in paper Figure 4A and Figure 6A. Consistent with the findings of Ross (2018), the N1P2 amplitudes of both onset and offset CAEPs were larger than the ACC responses, due to the tiny (close to the noise floor) or absence of ACC responses.

Regarding the N1P2 amplitude in experiment 2, a GLMrm analysis revealed significant effects of f_m, response type, and their interaction. The mean amplitude was 4.249/4.228/5.258/5.665 µV for f_m of 40/80/160/320 Hz, respectively. A significant difference between f_m was only observed for 80 Hz vs 160 Hz. Consistent with this, pairwise comparisons within each response type only showed a just significant smaller onset N1P2 amplitude in 80 Hz condition compared

to the 160 Hz condition (p = 0.048). The mean amplitude was 8.547/2.543/1.853/6.458 µV for onset/ACC1/ACC2/offset, respectively. Both onset and offset CAEPs were larger than the ACC responses. There were no significant differences between ACC1 and ACC2, and between onset and offset. Within each f_m, pairwise comparisons also showed no significant difference between onset and offset CAEPs, and between ACC1 and ACC2 responses (near noise floor). The onset and offset CAEPs were significantly larger than ACC responses, except for the comparison between ACC1 and offset for f_m = 80 Hz, and between ACC1 and offset, and ACC2 and offset for f_m = 160 Hz. Significant correlations were observed among modulation frequencies for all onset N1P2 amplitudes and for most offset N1P2 amplitudes, except for 320 vs 40, and 320 vs 160 Hz. The offset CAEPs were more correlated with the ACC responses than with the onset responses, mainly due to their small amplitudes.

For N1 latency, the GLMrm analysis showed no significant effect of either f_m or response type. The mean latency was 107/109/107/101 ms for onset/ACC1/ACC2/offset, and 107/105/104/109 ms for 40/80/160/320 Hz.

- CAEPs of experiment 3

Regarding the filtered clicks (paper Figure 4C and Figure 6C), there were no ACC2 responses (for inward IPTD changes) recorded in experiment 3. Overall, the N1P2 amplitude of both onset and offset responses increased with increasing pulse rates. Similarly to experiment 2, the ACC1 responses were either small (near the noise floor) or absent.

For N1P2 amplitude, GLMrm showed a significant effect of pulse rate, response type, and their interactions (p<0.01). The mean amplitude was 2.31/3.43/5.36/5.35 µV for 40/80/160/320 pps, respectively. There were no significant differences between pulse rates of 40 and 80 pps, and between 160 and 320 pps. Within each response type, pairwise comparisons showed no significant differences between pulse rates for both ACC1 and offset responses. For the onset CAEPs, there were significant differences between most pulse rates (p <0.01), except for conditions of 40 vs 80 pps, and 160 vs 320 pps. The mean amplitude was 5.48/2.52/4.34 µV for onset/ACC1/offset, and only the difference between onset and ACC1 responses was significant. Within each pulse rate, pairwise comparisons showed no significant differences between response types for most pulse rates, except that for 160 pps and 320 pps, there was a significantly larger onset N1P2 amplitude than the offset one (p=0.009, and p = 0.014). There were no correlations between N1P2 amplitudes of different pulse rates for both onset and offset responses.

For N1 latency, GLMrm revealed a significant effect of pulse rate, but not of response types or their interactions. The mean latency was 141/134/119/114 ms for 40/80/160/320 pps, respectively. The N1 latency was significantly shorter for 320 pps compared to 40 pps and 80 pps, and for 160 pps compared to 80 pps. Within each response type, pairwise comparison showed significant N1 latency differences only for 320 pps vs 40 pps, 320 pps vs 80, and 80 pps vs 160 pps for the onset CAEPs, and between 40 pps and 320 pps (p = 0.044) for ACC1. The mean latency was 131/129/120 ms for onset/ACC1/offset responses with no significant differences between them. Within each pulse rate, there were nearly no significant differences between the three response types, except that the onset N1 latency was significantly larger than the offset one (p = 0.011) for the 80 pps.

- Comparison CAEPs evoked with 40 Hz modulation rate stimuli

In experiment 3, we did not measure ACC2 data (inwards changes) for the filtered clicks, so only the onset, ACC1 (outwards changes), and offset responses of the five types of 40 Hz modulated SAM tones and the 40-pps filtered clicks were analyzed using GLMrm (with factors: stimuli type [400/800/1200/1600/4000SAM/40-pps-clicks], and response type [onset, ACC1 and offset]).
GLMrm showed a significant effect ($p<0.005$) of stimulus type, response type, and their interaction on the N1P2 amplitude. The mean amplitude was 7.544/6.271/5.860/4.759/5.046/2.309 µV for 400/800/1200/1600/4000SAM/40-pps-clicks, respectively. Pairwise comparison revealed significant differences between 400 Hz and 1600 Hz SAM tones, and between 40-pps filtered clicks and all four low-frequency SAM tones. Within each response type, the pairwise comparison showed that: 1) the onset response amplitude of the 40-pps filtered clicks was significantly smaller than most SAM tones except for the 800 Hz; 2) the 400 and 800 Hz SAM tones evoked significant larger responses than the other three SAM tones and the 40-pps filtered clicks for ACC1 response; 3) there were no significant differences among different stimulus types for the offset responses.
The GLMrm analysis revealed a significant effect of stimulus type, response type, as well as their interaction on N1 latency. The mean latency was 110/112/114/120/104/141ms for 400/800/1200/1600/4000SAM/40-pps-clicks. Pairwise comparison showed significant differences in latency between 1600 Hz and 4000 Hz SAM tones, and between 40-pps filtered clicks and the four low-frequency SAM tones. Further analysis within each response type showed that for onset responses, the 40-pps filtered clicks had a significantly different latency from most other stimulus types, except for the 1600 Hz SAM tones ($p = 0.051$). Within each stimulus type, there were no significant differences in latency among the three response types for both 4000 Hz SAM tones and 40-pps filtered clicks.

Time-frequency domain

Supplementary Figure 2A shows the time-frequency representations or wavelet spectrograms after wavelet transformations for stimuli with 40-Hz modulation frequency or 40-pps pulse rate in the frequency range of 2-50 Hz in the linear y-scale. The wavelet spectrograms reveal that the onset, ACC1, ACC2, and offset CAEPs are primarily dominated by frequencies below 30 Hz, while the 40-Hz ASSRs are centered around 40 Hz except during the 2s-silence period. Wavelet time-frequency visualization reveals some interactions between the transient CAEPs and ASSRs. A clear reset, represented by blue gaps in the 40 Hz regions, can be seen in the ASSRs whenever the P1-N1-P2 complex is detected and pronounced. This suggests that the transient CAEPs desynchronize the steady-state activity. For example, for f_c of 400, 800, and 1200 Hz, the ASSRs were suppressed or reset at approximately 0, 2, and 4 s, respectively, and the ASSRs in these time windows (T1, T2, T3) exhibit notable energy differences.

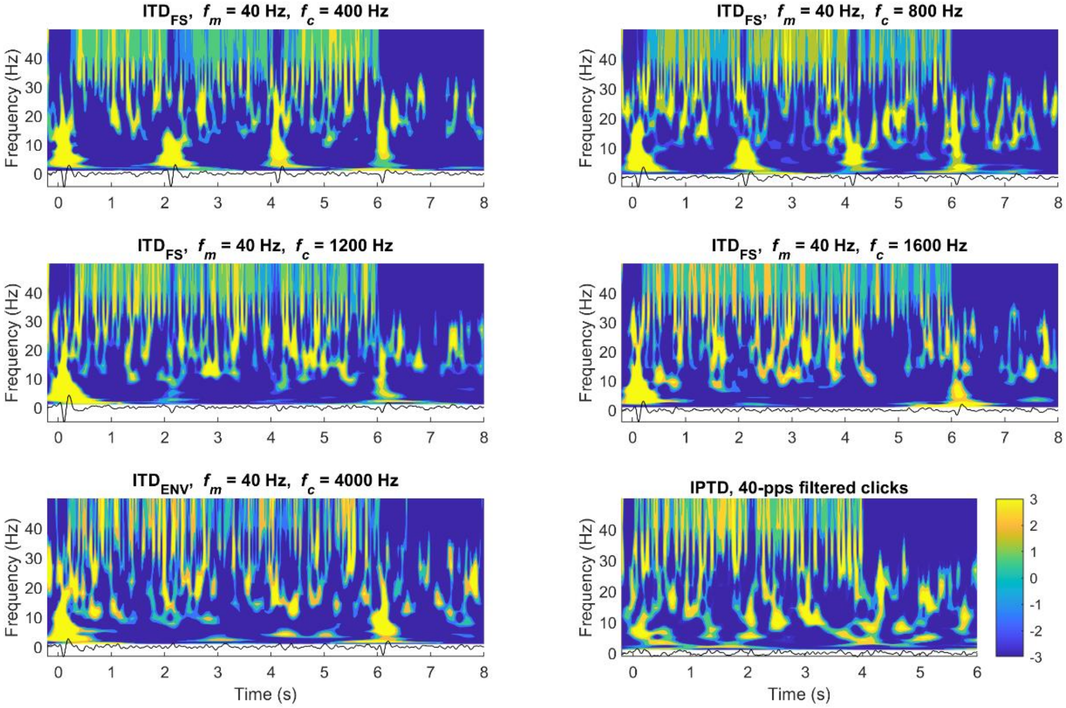

(A)

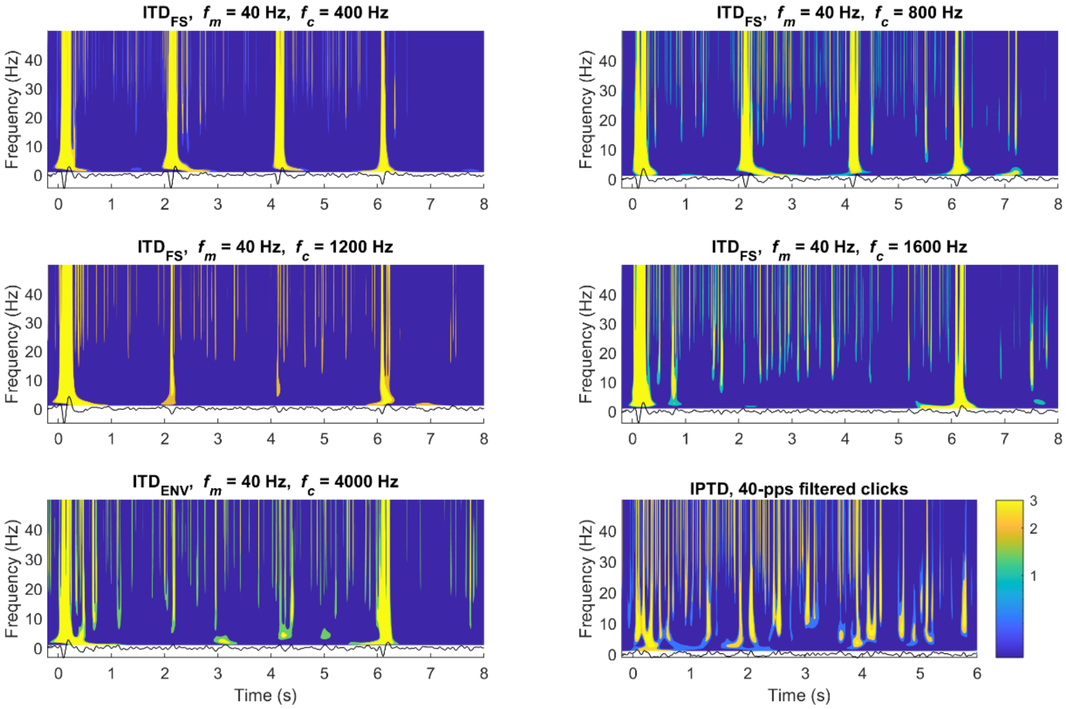

(B)

Supplementary Figure 2 The average response in the time-frequency domain for conditions with f_m= 40 Hz or pulse rate = 40 pps. (A) the number of cycles is $n = 6$, and (B) $n = 2$, with log colorscale. The time-frequency spectrum was obtained by applying wavelet analysis on the average response shown at the bottom of each panel.

Both Figure 8 and Supplementary Figure 2A have $n = 6$ cycles, which determines the temporal and spectral precision. An increase in n leads to decreased temporal precision but increased spectral precision, and vice versa. To prioritize temporal precision, Supplementary Figure 2B presents the same results as Supplementary Figure 2A but with $n = 2$ cycles. The color bar uses a log color scale for improved visualization. Compared to the ACC evoked by low-frequency ITDfs, Supplementary Figure 2B shows that the change responses evoked by the high-frequency ITDenv are much smaller and more similar to the surrounding brain activities, making it more challenging to determine the presence of ACC response.

Frequency domain (ASSRs)

A comparison between the 40-Hz ASSRs shown in Supplementary Figure 3 (A, the top-left panel of B and C, with the two parallel red dashed lines representing 30 and 50 Hz, respectively) and Supplementary Figure 2 reveals slight differences in different time durations. To examine possible differences in the steady state responses before and after interruption by the stimulus onset, ITD changes, and offset, the ASSRs in different time windows were analyzed.

Supplementary Figure 3 shows the overall average ASSRs across participants for different analysis windows (panels 1-5: T1, T2, T3, T4, T1234, or T124). The colored curves in the cream area are the average ASSR from each individual. The red, blue, black, and pink curves represent the group average ASSR across participants for various test conditions: (A) f_c= [400, 800, 1200, 1600] Hz; (B) f_m= [40, 80, 160, 320] Hz; (C) pulse rate = [40, 80, 160, 320] pps. The numbers with corresponding colors indicate the ASSR values at the modulation frequency within one of the analysis windows. The bottom right panel shows violin plots of the ASSR amplitude at the modulation frequency, within 8s (T1234) for the SAM tones or 6s (T124) for the filtered clicks.

In general, the 40-Hz ASSR decreased with increasing carrier frequency for the SAM tones. There was no ASSR in T4 (silence), the values shown are the noise floor around the modulation frequency, as expected.

Regarding experiment 1 (Supplementary Figure 3A), the overall mean 40-Hz ASSR amplitudes were 0.206/0.215/0.209/0.035/0.153µV within T1/T2/T3/T4/T1234, and 0.187/0.173/0.152/0.142 µV for 400/800/1200/1600 Hz. As found by Ross (2018), the amplitude of the 40-Hz ASSR within T1234 declined gradually with increasing carrier frequency. GLMrm (factors: analysis window, T1, T2, T3, T4, T1234; f_c) showed significant effect of f_c and analysis window, as well as their interaction. However, pairwise comparison showed no significant differences between different carrier frequencies. Pairwise comparision revealed significant differences between T4 (silence) and the other analysis windows (T1, T2, T3, T1234), as well as between T1234 and the other windows (T1, T2, T3, T4). Within each carrier frequency, the 40-Hz ASSR amplitudes were not significantly different among T1, T1, and T3.

The 40-Hz ASSR of different f_c SAM tones were all correlated, but there was no correlation between the f_c limit and either N1P2 or 40-Hz ASSR amplitude.

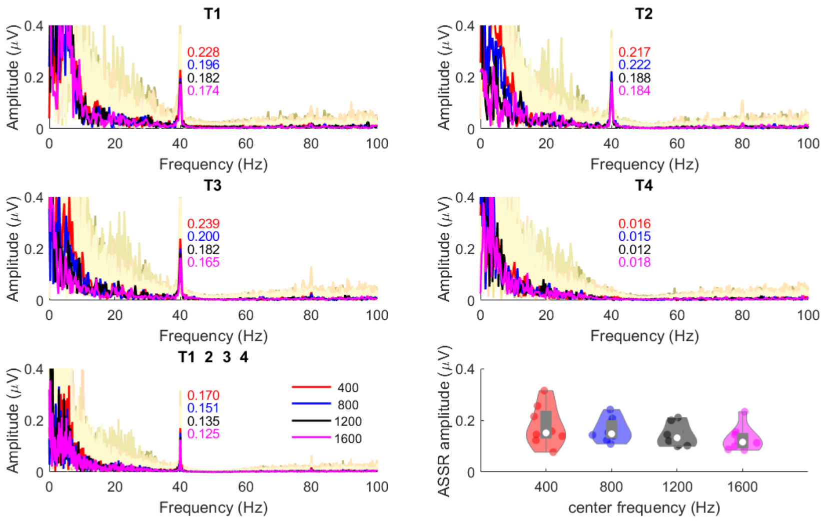
T1
0.228
0.196
0.182
0.174
T2
0.217
0.222
0.188
0.184
T3
0.239
0.200
0.182
0.165
T4
0.016
0.015
0.012
0.018
T1 2 3 4
0.170
0.151
0.135
0.125
400
800
1200
1600
Amplitude (μV)
Frequency (Hz)
ASSR amplitude (μV)
center frequency (Hz)

(A)

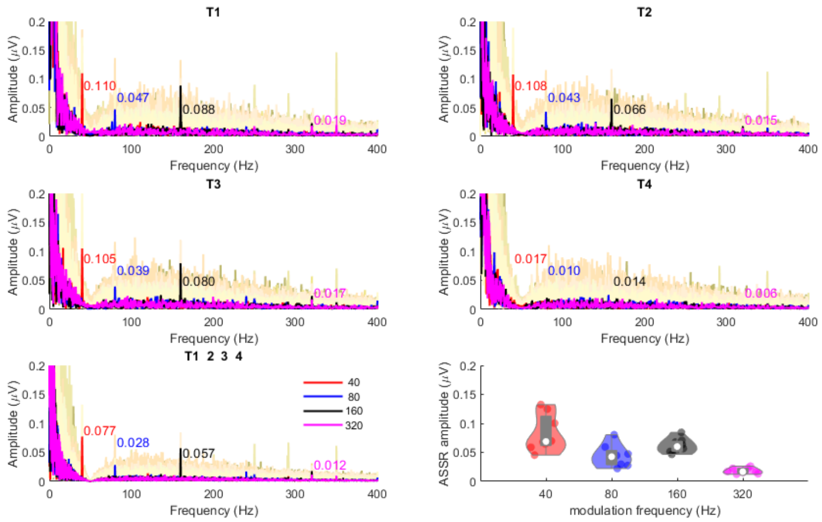
T1
0.110
0.047
0.088
0.019
T2
0.108
0.043
0.066
0.015
T3
0.105
0.039
0.080
0.017
T4
0.017
0.010
0.014
0.006
T1 2 3 4
0.077
0.028
0.057
0.012
40
80
160
320
Amplitude (μV)
Frequency (Hz)
ASSR amplitude (μV)
modulation frequency (Hz)

(B)

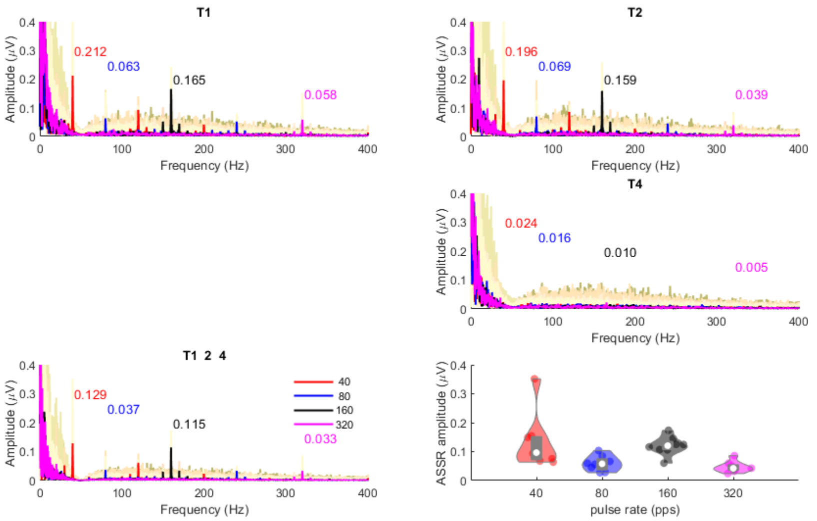

(C)

Supplementary Figure 3 The individual and group average ASSRs in the frequency domain. The red, blue, black, and pink spectrums are the overall average ASSR across participants for different test conditions: (A) f_c= [400, 800, 1200, 1600] Hz;. (B) f_m= [40, 80, 160, 320] Hz. (C) pulse rate = [40, 80, 160, 320] pps. All the other colored curves in the background are the average ASSR from each individual. The first five panels are the ASSRs in different analysis time windows (T1, T2, T3, T4, T1234, or T124). The numbers shown in different colors are the 40-Hz ASSR values within the corresponding time window for each carrier frequency. The bottom right panel shows the violin plots of the ASSR amplitude for each carrier frequency, with an analysis window of 8s (T1234) for SAM tones or 6s (T124) for the filtered clicks. The solid dots in each violin plot are individual ASSRs at the corresponding f_m or pulse rate of each participant.

Supplementary Figure 3B and C show the ASSRs at modulation rates of 40, 80, 160, and 320 Hz in various analysis time windows. The values in different colors indicate the corresponding ASSR values at different modulation rates. In general, the ASSRs of high carrier frequency stimuli are smaller than those of low carrier frequency (<=1600) SAM tones, and the ASSRs of filtered clicks are larger than those of high carrier frequency SAM tones. Among both types of high-frequency stimuli, the order is 40-Hz ASSR > 160-Hz ASSR > 80-Hz ASSR > 320-Hz ASSR. The larger 40-Hz ASSR evoked by the filtered clicks is mainly due to better phase locking to the envelope, as reported by Hu et al (2022).

Regarding experiment 2 (Supplementary Figure 3B), the overall mean ASSR amplitudes were 0.079/0.071/0.072/0.033/0.052 μV within T1/T2/T3/T4/T1234, and 0.093/0.058/0.071/0.022 μV for 40/80/160/320 Hz. GLMrm (factors: analysis window, T1, T2, T3, T4, T1234; f_m) showed significant effect of f_m and analysis window, as well as their interaction. Pairwise comparisons showed no significant differences for f_m 40 vs 80, 40 vs 160, 80 vs 160 Hz. Similar to experiment 1, pairwise comparisons showed significant differences between T4 (silence) and the other analysis windows (T1, T2, T3, T1234) as well as between T1234 and the other four analysis windows, both across modulation frequencies and within individual ones. Pairwise comparisons for each analysis widow showed that within T1, there were significant differences between most pulse rates except for 40 vs 160, 80 vs 160 pps; for T2, T3, T4, and T1234, the ASSR of 320 Hz was significantly smaller (T2, $p<0.01$; T3, T4, and T1234, $p<0.05$) than the other three modulation frequencies. There was no correlation between the f_m limit and the N1P2 amplitude or the ASSR amplitude. The onset responses of different modulation frequencies were all correlated with each other, and the offset N1P2 amplitudes of 40, 80, and 160 Hz were correlated with each other, but not with that of 320 Hz.

For experiment 3 (Supplementary Figure 3C), the mean ASSR amplitudes were 0.143/0.131/0.032/0.089 µV in T1/T2/T4/T124, and 0.148/0.075/0.123/0.048 µV for 40/80/160/320 pps. GLMrm (factors: analysis window, T1, T2, T4, T124; f_m) showed significant effect of f_m and analysis window, as well as their interaction. Pairwise comparison only showed significant ASSR amplitude differences for 160 vs 80, and 160 vs 320 pps. Unlike experiments 1 and 2, the pairwise comparison showed significant differences among all the analysis windows. Specifically, the 160 pps condition evoked a significantly larger ASSR compared to the 80 and 320 pps conditions within both T1 and T124, as well as the 320 pps condition within T2. Additionally, the noise floor in T4 (silence) was significantly smaller for 320 pps compared to the other three pulse rates. No correlation was found between the pulse rate upper limit and the N1P2 amplitude or the ASSR amplitude. However, the ASSR amplitude of the 40 pps condition was correlated with the 160 pps condition.
